# *N*′-(2-Chloro­benzyl­idene)-2-fluoro­benzohydrazide

**DOI:** 10.1107/S1600536810053043

**Published:** 2010-12-24

**Authors:** Wei-Guang Zhang

**Affiliations:** a College of Chemistry and Chemical Engineering, Qiqihar University, Qiqihar 161006, People’s Republic of China

## Abstract

The title hydrazone compound, C_14_H_10_ClFN_2_O, adopts an *E* configuration about the C=N double bond. The dihedral angle between the two substituted benzene rings is 11.6 (2)°. The F atom is disordered over two sites with occupancies of 0.488 (2) and 0.512 (2). In the crystal, mol­ecules are linked through inter­molecular N—H⋯O hydrogen bonds, forming chains along the *a* axis. C—H⋯F and C—H⋯O inter­actions also occur.

## Related literature

For the biological properties of hydrazone compounds, see: Ajani *et al.* (2010 ▶); Angelusiu *et al.* (2010 ▶); Zhang *et al.* (2010 ▶); Horiuchi *et al.* (2009 ▶). For the crystal structures of hydrazone compounds, see: Ban (2010 ▶); Hussain *et al.* (2010 ▶); Shalash *et al.* (2010 ▶); Khaledi *et al.* (2009 ▶).
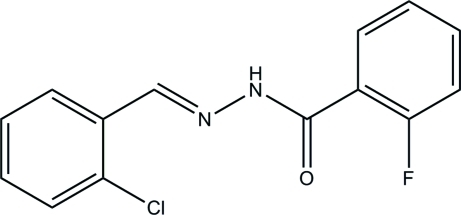


## Experimental

### 

#### Crystal data

C_14_H_10_ClFN_2_O
*M* *_r_* = 276.69
Monoclinic, 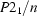

*a* = 7.1110 (14) Å
*b* = 25.291 (3) Å
*c* = 7.6560 (15) Å
β = 111.472 (3)°
*V* = 1281.3 (4) Å^3^
*Z* = 4
Mo *K*α radiation
μ = 0.30 mm^−1^
*T* = 298 K
0.20 × 0.17 × 0.17 mm

#### Data collection

Bruker APEXII diffractometer
Absorption correction: multi-scan (*SADABS*; Sheldrick, 1996 ▶) *T*_min_ = 0.942, *T*_max_ = 0.950
10805 measured reflections
2734 independent reflections
1771 reflections with *I* > 2σ(*I*)
*R*_int_ = 0.050

#### Refinement

*R*[*F* ^2^ > 2σ(*F* ^2^)] = 0.053
*wR*(*F* ^2^) = 0.123
*S* = 1.03
2734 reflections
185 parameters
3 restraints
H atoms treated by a mixture of independent and constrained refinement
Δρ_max_ = 0.20 e Å^−3^
Δρ_min_ = −0.17 e Å^−3^

### 

Data collection: *APEX2* (Bruker, 2007 ▶); cell refinement: *SAINT* (Bruker, 2007 ▶); data reduction: *SAINT*; program(s) used to solve structure: *SHELXS97* (Sheldrick, 2008 ▶); program(s) used to refine structure: *SHELXL97* (Sheldrick, 2008 ▶); molecular graphics: *SHELXTL* (Sheldrick, 2008 ▶); software used to prepare material for publication: *SHELXTL*.

## Supplementary Material

Crystal structure: contains datablocks global, I. DOI: 10.1107/S1600536810053043/om2388sup1.cif

Structure factors: contains datablocks I. DOI: 10.1107/S1600536810053043/om2388Isup2.hkl

Additional supplementary materials:  crystallographic information; 3D view; checkCIF report

## Figures and Tables

**Table 1:** Hydrogen-bond geometry (Å, °)

*D*—H⋯*A*	*D*—H	H⋯*A*	*D*⋯*A*	*D*—H⋯*A*
N2—H2⋯O1^i^	0.86 (2)	2.07 (2)	2.912 (2)	167 (2)
C3—H3⋯F1*A*^ii^	0.93	2.40	3.259 (2)	154 (2)
C7—H7⋯O1^i^	0.93	2.50	3.270 (2)	140 (2)

Symmetry codes: (i) 
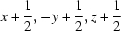
; (ii) 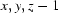.

**Fig. 1.**
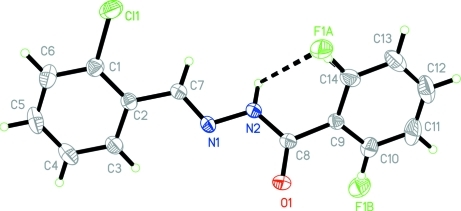
The molecular structure of the title compound, showing the atom-numbering scheme and displacement ellipsoids drawn at the 30% probability level.

**Fig. 2.**
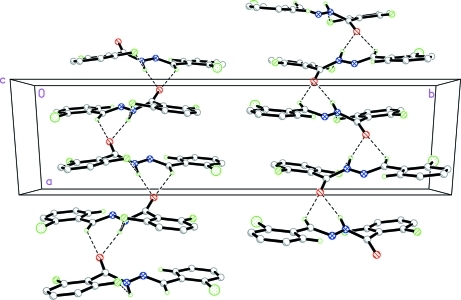
The molecular packing of the title compound viewed along the c axis. Hydrogen bonds are shown as dashed lines. H-atoms not involved in hydrogen bonding have been omitted for clarity.

## supplementary crystallographic information

### Comment

Benzoylhydrazones are a kind of special Schiff base bearing the –C(O)—NH—N=CH– groups. The hydrazone compounds have received much attention for their excellent biological properties (Ajani *et al.*, 2010; Angelusiu *et al.*, 2010; Zhang *et al.*, 2010; Horiuchi *et al.*, 2009) as well as their crystal structures (Ban, 2010; Hussain *et al.*, 2010; Shalash *et al.*, 2010; Khaledi *et al.*, 2009). In the present paper, the title new hydrazone compound is reported.

The compound adopts an *E* configuration about the C═N double bond (Fig. 1). The dihedral angle between the two substituted benzene rings is 11.6 (2)°. The F atom is disordered over two sites with occupancies of 0.488 (2) and 0.512 (2). There is an intramolecular N—H···F hydrogen bond in the molecule. In the crystal structure, molecules are linked through intermolecular N—H···O and C—H···O hydrogen bonds (Table 1), forming chains along the *a* axis (Fig. 2). Moreover, there still presence of one non-classical C—H···F hydrogen bonding (Table 1), and one weak pi-pi interaction with centroid-centroid distance of 3.712 (2) Å.

### Experimental

2-Chlorobenzaldehyde (0.140 g, 1 mmol) and 2-fluorobenzohydrazide (0.154 g, 1 mmol) were mixed in 50 ml me thanol. The mixture was stirred and refluxed for 30 min and cooled to room temperature to give a colorless solution. Colorless block-shaped single crystals were obtained on slow evaporation of the solution in air.

### Refinement

H2 was located in a difference Fourier map and refined with the N—H distance restrained to 0.86 (1) Å. The remaining H atoms were positioned geometrically, with C—H = 0.93 Å, and with *U*_iso_(H) = 1.2*U*_eq_(C). The F atom is disordered over two sites with occupancies of 0.512 (2) and 0.488 (2). The C-F distance was restrained (DFIX) to a target value of 1.350 (5) Å.

### Crystal data

**Table:**

C_14_H_10_ClFN_2_O	*F*(000) = 568
*M**_r_* = 276.69	*D*_x_ = 1.434 Mg m^−^^3^
Monoclinic, *P*2_1_/*n*	Mo *K*α radiation, λ = 0.71073 Å
Hall symbol: -P 2yn	Cell parameters from 1387 reflections
*a* = 7.1110 (14) Å	θ = 2.5–24.6°
*b* = 25.291 (3) Å	µ = 0.30 mm^−^^1^
*c* = 7.6560 (15) Å	*T* = 298 K
β = 111.472 (3)°	Block, colorless
*V* = 1281.3 (4) Å^3^	0.20 × 0.17 × 0.17 mm
*Z* = 4	

### Data collection

**Table:**

Bruker APEXII diffractometer	2734 independent reflections
Radiation source: fine-focus sealed tube	1771 reflections with *I* > 2σ(*I*)
graphite	*R*_int_ = 0.050
ω scans	θ_max_ = 27.0°, θ_min_ = 1.6°
Absorption correction: multi-scan (*SADABS*; Sheldrick, 1996)	*h* = −8→9
*T*_min_ = 0.942, *T*_max_ = 0.950	*k* = −32→32
10805 measured reflections	*l* = −9→9

### Refinement

**Table:**

Refinement on *F*^2^	Primary atom site location: structure-invariant direct methods
Least-squares matrix: full	Secondary atom site location: difference Fourier map
*R*[*F*^2^ > 2σ(*F*^2^)] = 0.053	Hydrogen site location: inferred from neighbouring sites
*wR*(*F*^2^) = 0.123	H atoms treated by a mixture of independent and constrained refinement
*S* = 1.03	*w* = 1/[σ^2^(*F*_o_^2^) + (0.0447*P*)^2^ + 0.2578*P*] where *P* = (*F*_o_^2^ + 2*F*_c_^2^)/3
2734 reflections	(Δ/σ)_max_ < 0.001
185 parameters	Δρ_max_ = 0.20 e Å^−^^3^
3 restraints	Δρ_min_ = −0.17 e Å^−^^3^

### Special details

**Table:**

Geometry. All e.s.d.'s (except the e.s.d. in the dihedral angle between two l.s. planes) are estimated using the full covariance matrix. The cell e.s.d.'s are taken into account individually in the estimation of e.s.d.'s in distances, angles and torsion angles; correlations between e.s.d.'s in cell parameters are only used when they are defined by crystal symmetry. An approximate (isotropic) treatment of cell e.s.d.'s is used for estimating e.s.d.'s involving l.s. planes.
Refinement. Refinement of *F*^2^ against ALL reflections. The weighted *R*-factor *wR* and goodness of fit *S* are based on *F*^2^, conventional *R*-factors *R* are based on *F*, with *F* set to zero for negative *F*^2^. The threshold expression of *F*^2^ > σ(*F*^2^) is used only for calculating *R*-factors(gt) *etc*. and is not relevant to the choice of reflections for refinement. *R*-factors based on *F*^2^ are statistically about twice as large as those based on *F*, and *R*- factors based on ALL data will be even larger.

### Fractional atomic coordinates and isotropic or equivalent isotropic displacement parameters (Å^2^)

**Table:**

	*x*	*y*	*z*	*U*_iso_*/*U*_eq_	Occ. (<1)
Cl1	0.79975 (13)	0.45321 (3)	0.51687 (11)	0.0759 (3)	
F1A	0.6784 (6)	0.24779 (12)	0.8540 (4)	0.0821 (14)	0.512 (4)
F1B	0.7132 (5)	0.09899 (12)	0.5057 (4)	0.0719 (13)	0.488 (4)
O1	0.5332 (3)	0.19241 (6)	0.3381 (2)	0.0543 (5)	
N1	0.6941 (3)	0.29004 (7)	0.3616 (2)	0.0414 (5)	
N2	0.7342 (3)	0.25676 (8)	0.5135 (3)	0.0445 (5)	
H2	0.826 (3)	0.2667 (9)	0.617 (3)	0.053*	
C1	0.7557 (4)	0.42979 (10)	0.2922 (3)	0.0478 (6)	
C2	0.7356 (3)	0.37588 (9)	0.2559 (3)	0.0401 (5)	
C3	0.7001 (4)	0.35952 (10)	0.0726 (3)	0.0489 (6)	
H3	0.6845	0.3237	0.0435	0.059*	
C4	0.6877 (4)	0.39541 (13)	−0.0655 (4)	0.0642 (8)	
H4	0.6663	0.3839	−0.1866	0.077*	
C5	0.7070 (5)	0.44840 (13)	−0.0241 (4)	0.0753 (9)	
H5	0.6971	0.4727	−0.1183	0.090*	
C6	0.7407 (4)	0.46597 (11)	0.1538 (4)	0.0663 (8)	
H6	0.7532	0.5019	0.1808	0.080*	
C7	0.7584 (3)	0.33724 (9)	0.4035 (3)	0.0416 (6)	
H7	0.8200	0.3470	0.5288	0.050*	
C8	0.6477 (3)	0.20886 (9)	0.4899 (3)	0.0387 (5)	
C9	0.6963 (3)	0.17703 (9)	0.6648 (3)	0.0375 (5)	
C10	0.7213 (4)	0.12300 (10)	0.6606 (4)	0.0507 (6)	
H10	0.7113	0.1071	0.5481	0.061*	0.512 (4)
C11	0.7599 (4)	0.09235 (12)	0.8154 (5)	0.0741 (9)	
H11	0.7775	0.0561	0.8081	0.089*	
C12	0.7729 (4)	0.11456 (17)	0.9811 (5)	0.0805 (11)	
H12	0.7987	0.0934	1.0867	0.097*	
C13	0.7482 (4)	0.16775 (16)	0.9932 (4)	0.0712 (9)	
H13	0.7566	0.1833	1.1059	0.085*	
C14	0.7105 (4)	0.19780 (11)	0.8342 (3)	0.0533 (7)	
H14	0.6938	0.2341	0.8423	0.064*	0.488 (4)

### Atomic displacement parameters (Å^2^)

**Table:**

	*U*^11^	*U*^22^	*U*^33^	*U*^12^	*U*^13^	*U*^23^
Cl1	0.1118 (7)	0.0547 (5)	0.0717 (6)	−0.0195 (4)	0.0461 (5)	−0.0190 (4)
F1A	0.137 (3)	0.059 (2)	0.068 (2)	−0.020 (2)	0.059 (2)	−0.0216 (16)
F1B	0.090 (3)	0.046 (2)	0.082 (3)	0.0040 (16)	0.034 (2)	−0.0095 (17)
O1	0.0658 (12)	0.0487 (10)	0.0334 (10)	−0.0124 (9)	0.0006 (8)	−0.0030 (8)
N1	0.0445 (11)	0.0424 (11)	0.0313 (10)	−0.0007 (9)	0.0066 (8)	0.0066 (9)
N2	0.0504 (13)	0.0412 (11)	0.0303 (11)	−0.0095 (9)	0.0011 (9)	0.0026 (9)
C1	0.0509 (15)	0.0456 (15)	0.0503 (16)	−0.0004 (12)	0.0224 (12)	0.0004 (12)
C2	0.0349 (13)	0.0450 (14)	0.0387 (13)	0.0013 (10)	0.0115 (10)	0.0033 (11)
C3	0.0513 (15)	0.0525 (15)	0.0407 (15)	0.0063 (12)	0.0141 (12)	0.0026 (12)
C4	0.0694 (19)	0.083 (2)	0.0387 (16)	0.0155 (16)	0.0182 (14)	0.0127 (15)
C5	0.089 (2)	0.077 (2)	0.065 (2)	0.0196 (18)	0.0336 (18)	0.0363 (18)
C6	0.080 (2)	0.0455 (16)	0.080 (2)	0.0077 (14)	0.0365 (18)	0.0146 (15)
C7	0.0449 (14)	0.0424 (14)	0.0332 (13)	−0.0030 (11)	0.0093 (10)	0.0003 (11)
C8	0.0381 (13)	0.0418 (13)	0.0332 (13)	−0.0006 (11)	0.0095 (11)	−0.0022 (10)
C9	0.0318 (12)	0.0447 (14)	0.0314 (12)	−0.0034 (10)	0.0062 (9)	0.0026 (10)
C10	0.0434 (15)	0.0472 (15)	0.0591 (17)	0.0026 (12)	0.0161 (13)	0.0076 (14)
C11	0.0605 (19)	0.0587 (19)	0.096 (3)	0.0067 (15)	0.0203 (18)	0.0356 (19)
C12	0.057 (2)	0.106 (3)	0.066 (2)	−0.0101 (18)	0.0085 (16)	0.044 (2)
C13	0.0576 (18)	0.117 (3)	0.0361 (16)	−0.0247 (18)	0.0141 (13)	0.0039 (17)
C14	0.0479 (15)	0.0685 (19)	0.0409 (16)	−0.0120 (13)	0.0131 (12)	−0.0060 (14)

### Geometric parameters (Å, °)

**Table:**

Cl1—C1	1.736 (3)	C5—C6	1.368 (4)
F1A—C14	1.303 (3)	C5—H5	0.9300
F1B—C10	1.315 (3)	C6—H6	0.9300
O1—C8	1.222 (2)	C7—H7	0.9300
N1—C7	1.276 (3)	C8—C9	1.491 (3)
N1—N2	1.379 (2)	C9—C14	1.368 (3)
N2—C8	1.340 (3)	C9—C10	1.380 (3)
N2—H2	0.861 (16)	C10—C11	1.357 (4)
C1—C6	1.374 (3)	C10—H10	0.9300
C1—C2	1.388 (3)	C11—C12	1.360 (4)
C2—C3	1.395 (3)	C11—H11	0.9300
C2—C7	1.457 (3)	C12—C13	1.364 (5)
C3—C4	1.372 (3)	C12—H12	0.9300
C3—H3	0.9300	C13—C14	1.376 (4)
C4—C5	1.372 (4)	C13—H13	0.9300
C4—H4	0.9300	C14—H14	0.9300
			
C7—N1—N2	114.48 (18)	O1—C8—N2	123.3 (2)
C8—N2—N1	119.66 (18)	O1—C8—C9	121.7 (2)
C8—N2—H2	123.0 (16)	N2—C8—C9	114.99 (19)
N1—N2—H2	116.9 (16)	C14—C9—C10	115.9 (2)
C6—C1—C2	121.7 (2)	C14—C9—C8	123.8 (2)
C6—C1—Cl1	118.2 (2)	C10—C9—C8	120.2 (2)
C2—C1—Cl1	120.10 (19)	F1B—C10—C11	116.9 (3)
C1—C2—C3	117.4 (2)	F1B—C10—C9	121.0 (3)
C1—C2—C7	122.0 (2)	C11—C10—C9	122.1 (3)
C3—C2—C7	120.6 (2)	C11—C10—H10	118.9
C4—C3—C2	121.1 (2)	C9—C10—H10	118.9
C4—C3—H3	119.5	C10—C11—C12	120.1 (3)
C2—C3—H3	119.5	C10—C11—H11	119.9
C3—C4—C5	119.7 (3)	C12—C11—H11	119.9
C3—C4—H4	120.1	C11—C12—C13	120.3 (3)
C5—C4—H4	120.1	C11—C12—H12	119.9
C6—C5—C4	120.9 (3)	C13—C12—H12	119.9
C6—C5—H5	119.6	C12—C13—C14	118.2 (3)
C4—C5—H5	119.6	C12—C13—H13	120.9
C5—C6—C1	119.2 (3)	C14—C13—H13	120.9
C5—C6—H6	120.4	F1A—C14—C9	121.8 (3)
C1—C6—H6	120.4	F1A—C14—C13	114.8 (3)
N1—C7—C2	120.3 (2)	C9—C14—C13	123.3 (3)
N1—C7—H7	119.9	C9—C14—H14	118.3
C2—C7—H7	119.9	C13—C14—H14	118.3

### Hydrogen-bond geometry (Å, °)

**Table:**

*D*—H···*A*	*D*—H	H···*A*	*D*···*A*	*D*—H···*A*
N2—H2···O1^i^	0.86 (2)	2.07 (2)	2.912 (2)	167 (2)
N2—H2···F1A	0.86 (2)	2.45 (2)	2.785 (2)	104 (2)
C3—H3···F1A^ii^	0.93	2.40	3.259 (2)	154 (2)
C7—H7···O1^i^	0.93	2.50	3.270 (2)	140 (2)

Symmetry codes: (i) *x*+1/2, −*y*+1/2, *z*+1/2; (ii) *x*, *y*, *z*−1.
